# Production of enterodiol from defatted flaxseeds through biotransformation by human intestinal bacteria

**DOI:** 10.1186/1471-2180-10-115

**Published:** 2010-04-16

**Authors:** Cheng-Zhi Wang, Xiao-Qing Ma, Dong-Hui Yang, Zhi-Rong Guo, Gui-Rong Liu, Ge-Xin Zhao, Jie Tang, Ya-Nan Zhang, Miao Ma, Shao-Qing Cai, Bao-Shan Ku, Shu-Lin Liu

**Affiliations:** 1 Department of Microbiology, Peking University Health Science Center, Beijing 100191, China; 2 Department of Natural Medicines, School of Pharmaceutical Sciences, Peking University Health Science Center, Beijing 100191, China; 3 Genomics Research Center (one of The State-Province Key Laboratories of Biomedicine-Pharmaceutics of China), Harbin Medical University, Harbin 150081, China; 4 Department of Pharmacology, Peking University Health Science Center, Beijing, 100191, China; 5 Department of Microbiology and Infectious Diseases, University of Calgary, Calgary, AB, T2N 4N1, Canada

## Abstract

**Background:**

The effects of enterolignans, e.g., enterodiol (END) and particularly its oxidation product, enterolactone (ENL), on prevention of hormone-dependent diseases, such as osteoporosis, cardiovascular diseases, hyperlipemia, breast cancer, colon cancer, prostate cancer and menopausal syndrome, have attracted much attention. To date, the main way to obtain END and ENL is chemical synthesis, which is expensive and inevitably leads to environmental pollution. To explore a more economic and eco-friendly production method, we explored biotransformation of enterolignans from precursors contained in defatted flaxseeds by human intestinal bacteria.

**Results:**

We cultured fecal specimens from healthy young adults in media containing defatted flaxseeds and detected END from the culture supernatant. Following selection through successive subcultures of the fecal microbiota with defatted flaxseeds as the only carbon source, we obtained a bacterial consortium, designated as END-49, which contained the smallest number of bacterial types still capable of metabolizing defatted flaxseeds to produce END. Based on analysis with pulsed field gel electrophoresis, END-49 was found to consist of five genomically distinct bacterial lineages, designated Group I-V, with Group I strains dominating the culture. None of the individual Group I-V strains produced END, demonstrating that the biotransformation of substrates in defatted flaxseeds into END is a joint work by different members of the END-49 bacterial consortium. Interestingly, Group I strains produced secoisolariciresinol, an important intermediate of END production; 16S rRNA analysis of one Group I strain established its close relatedness with *Klebsiella*. Genomic analysis is under way to identify all members in END-49 involved in the biotransformation and the actual pathway leading to END-production.

**Conclusion:**

Biotransformation is a very economic, efficient and environmentally friendly way of mass-producing enterodiol from defatted flaxseeds.

## Background

Early in the 1980s, enterodiol (END) and enterolactone (ENL) were first detected in the serum, urine and bile of humans and several animals [1,2]. They were classified as phytoestrogens due to their origins from plants and their estrogenic as well as antiestrogenic activities in humans. Epidemiologic and pharmacologic studies have shown that END and particularly its oxidation product ENL have preventive effects on osteoporosis, cardiovascular diseases, hyperlipemia, breast cancer, colon cancer, prostate cancer and menopausal syndrome [3-7]. Unlike other plant-derived lignans, they are also known as mammalian lignan or enterolignan, because they are mainly found in mammals. Numerous studies have indicated that END and ENL can be produced from several plants, such as flaxseed, by bacteria in the intestinal tract of humans and animals. Thompson et al. tested 68 common plant foods and found that flaxseed flour and its defatted meal produced the highest yield of END and ENL *in vitro*, up to 800 times higher than that from others [8].

Flaxseed is the dried seed of *Linum usitatissimum *L., which is widely distributed in northern China, with an annual output of 420,000 tons (ranking fourth in the world). The important precursors of END and ENL synthesis include secoisolariciresinol diglucoside (SDG), secoisolariciresinol (SECO), matairesinol (MAT), lariciresinol (LCS) and pinoresinol (PRS) [9-11]. Among these precursors, SDG is the most abundant lignan in flaxseed, with a content of around 6.1-13.3 mg g^-1 ^(dry matter) in whole flaxseeds, and 11.7-24.1 mg g^-1 ^(dry matter) in the defatted flour [12].

Although *de novo *synthesis of END and ENL has been reported [13], the processes of synthesis are very complex and expensive, requiring more than ten major steps. More importantly, the reagents used in the reactions for the synthesis include LiAlH_4_, MeOH and several other chemicals, which are toxic and harmful to the environment. Therefore, biotransformation of precursors in plants to END or ENL is highly desirable.

**Figure 1:**
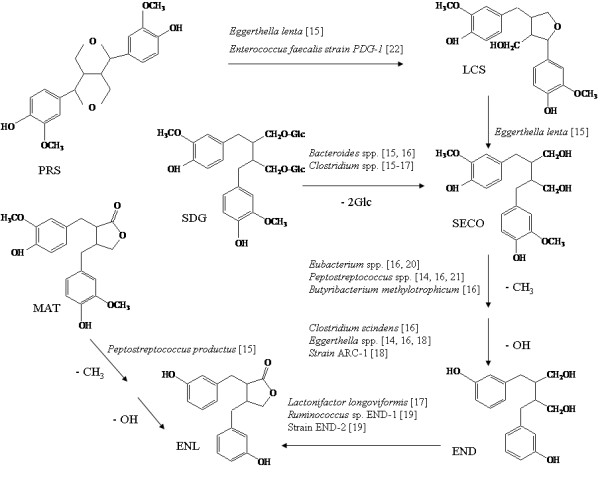
**Biotransformation pathway of END and ENL from plant-derived lignan SDG; bacteria that work at different steps of the pathway, along with the authors who reported them, are indicated**.

Biotransformation of SDG to END and ENL by human intestinal bacteria has been extensively studied, the pathway consisting of glycoside hydrolysis, demethylation, and dehydroxylation of SDG and its intermediates [9]. Bacteria that can produce END and ENL on plant lignans under strictly anaerobic conditions have been isolated from human feces [14-23] (Fig. 1). However, sufficient yields for marketing scale production of END and ENL by these microbes have not been achieved, largely due to the difficulty to create and maintain the strictly anaerobic culture conditions under which the bacteria can grow and conduct the biotransformation.

In China, flaxseeds are mainly used as oil crop. The defatted waste, though a rich source of lignans, is mostly used as animal feed. To establish a method for producing enterolignans from defatted flaxseeds by bacterial biotransformation, we screened human fecal samples and obtained cultures that can efficiently produce END. After 49 rounds of selection by successive subcultures of human fecal bacterial microbiota in media containing defatted flaxseeds as the only carbon source, we obtained a group of mixed bacteria that could metabolize flaxseeds to produce END under both anaerobic and aerobic culture conditions. In this paper, we report the method and discuss its potential applications for large scale production of enterolignans.

## Results

### Determining culture media for bacterial production of END from defatted flaxseeds

To select the human intestinal bacteria that could efficiently metabolize flaxseed lignans to produce END without the need of strictly anaerobic culture conditions, we compared three types of culture media (A, B, C; see components in Methods).

**Figure 2:**
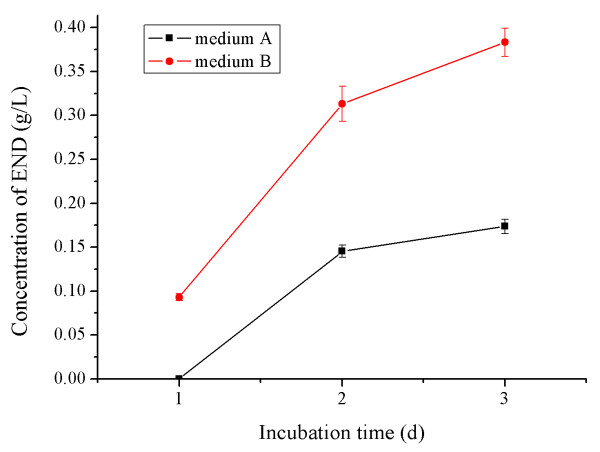
**END production curve in medium A and medium B**. Each data point represents the mean of at least 2 independent determinations. No END was detected in medium C.

When the cultures were terminated, END could be detected in media A and B, but not C, with the yield of END in medium B being considerably higher than that in medium A (Fig. 2). These results indicated that a nitrogen source (NH_4_Cl in this study, present in B but not in C) was necessary to support the bacteria that could transform flaxseed lignans into END. Based on these results, we chose medium B for bacterial cultures.

### Optimization of culture conditions for large-scale production of END

For large-scale production of END, we increased the volume of medium B from 3 ml to 2 liter with 40 g defatted flaxseeds in 4 liter Erlenmeyer flasks. In one of the Erlenmeyer flasks, 50 ml liquid paraffin was added on top of the culture medium; in another Erlenmeyer flask, no liquid paraffin was added, for comparison of effects of anaerobic *vs *aerobic culture conditions on END production. The culture was continued at 37°C for 6 days and then terminated for analysis of END production. Interestingly, cultures with or without liquid paraffin added on top of the culture had similar yields of END and the concentration of END reached 86.76 ± 4.19 mg l^-1 ^in both cases, demonstrating that biotransformation of flaxseed lignans into END in our system did not require strict anaerobic conditions.

### Enrichment of END

**Figure 3:**
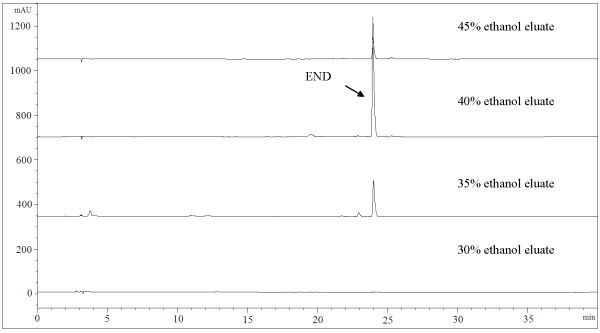
**HPLC elution profiles of END at different ethanol concentrations on XAD-2 resin; END was most efficiently eluted at 40% ethanol**.

We treated the cultures (in medium B; see above) with 3 fold volumes of 95% ethanol to terminate the culture and to precipitate the macromolecule substances in the culture. We then evaporated the supernatant at 50°C under reduced pressure and retrieved a ca. 30 g pellet from a 2 liter culture. We dissolved the pellet in 300 ml of 5% ethanol, chromatographed the solution on 300 g of XAD-2 macroporous resin column, and successively eluted the column with 2.5 liter of 5%-50% ethanol solutions, with 5% ethanol concentration gradient increases. Each elute was analyzed by HPLC. As shown in Fig. 3, END was mainly eluted by 40% ethanol; the END production could reach up to 3.9 mg g^-1^. The produced END was identified as (+)-END with reference to the published data ([α]^25^_D _+13° (c = 0.10, MeOH); [18]).

### Selection of END-producing bacteria by successive subcultures

**Figure 4:**
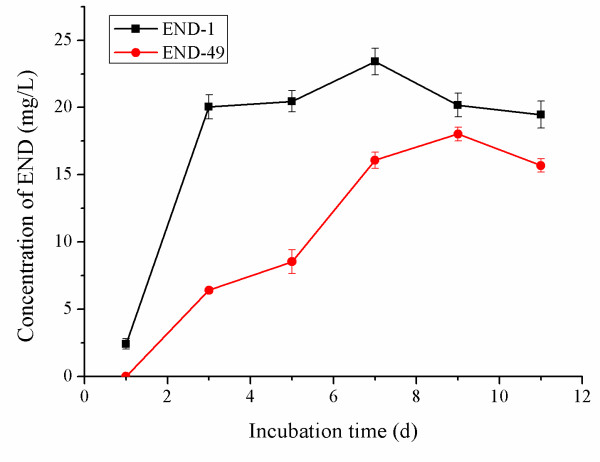
**Comparison of time courses of END production between END-1 and END-49**. Each data point represents the mean of at least 2 independent determinations.

In the first few passages, there was a great diversity of microbes in the culture as examined by Gram staining and PFGE analysis (data not shown). Starting with passage 40 (END-40), the microbial diversity became gradually reduced. END-49 was the last passage that could still produce END, although the product took a longer time to appear (Fig. 4) and the yield was also significantly decreased (18.0 ± 0.51 mg ml^-1 ^as compared to 23.42 ± 0.99 mg ml^-1 ^in END-1; p < 0.01). When END-49 was diluted for further passages, END was hardly detected. Therefore, we speculated that END-49 contained the minimal number of bacterial members that would be necessary to cooperate in producing END.

### Pulsed field gel electrophoresis (PFGE) analysis of END-49

**Figure 5:**
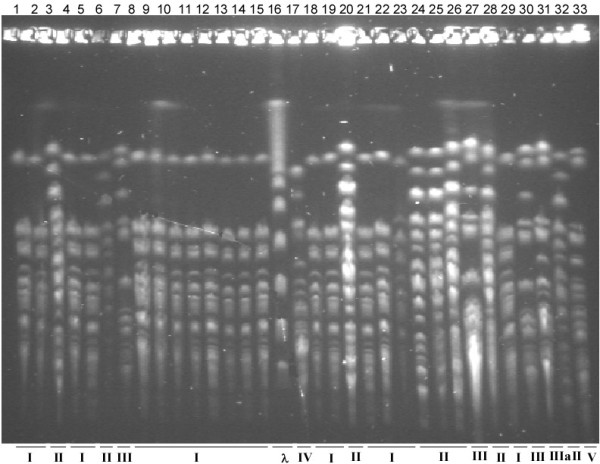
**PFGE patterns of SpeI-cleaved genomic DNA of 32 pure cultures obtained from END-49**. Assignment of the bacterial strains to Genome Group I, II, III, IV or V was indicated at the bottom of the PFGE photo.

A 0.1 ml aliquot of the END-49 culture was spread on an LB plate and well isolated single colonies were picked up the following day. We then took 32 colonies with seemingly different morphologies and isolated genomic DNA from them for PFGE analysis. Based on their similarities of PFGE patterns with SpeI cleavage, we categorized the 32 bacterial strains into five distinct groups (Group I - V), with Group I containing as many as 18 of the 32 strains (Fig. 5). The remaining 14 strains were categorized into four groups (group II - V; Fig. 5).

### Phylogenetic characterization of Group I strains

**Figure 6:**
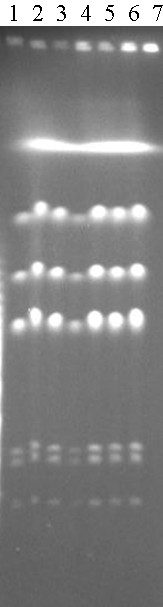
**PFGE patterns of I-CeuI cleaved genomic DNA of Genome Group I bacterial strains**. Lanes: 1, S1; 2, S2; 3, S3; 4, S4; 5, S5; 6, S6; 7, S7.

The dominance of Group I strains in the minimal bacterial consortium that was still capable of producing END from defatted flaxseeds suggests that this bacterial lineage might be the main player in the biotransformation to produce END. To assess their roles in this biochemical process, we randomly picked seven Group I colonies (designated S1 to S7), grew them on defatted flaxseeds and analyzed the culture for the presence of END. No END was detected from any of the seven Group I strains. Instead, we detected SECO, a key intermediate in the transformation of flaxseed lignans (e.g., SDG) to END (see figure 1), from all seven tested Group I strains. After one day of incubation, SECO concentration was 34.97 ± 0.98 mg l ^-1^. When the incubation continued, the maximum concentration reached 122.05 ± 7.67 mg l^-1^. No END or SECO was detected from the Group II-V strains. We initiated genomic analysis of these bacteria, beginning with S1 through S7, using the endonuclease I-CeuI, which reflects phylogenetic relationships among bacteria [24-26]. All seven strains had indistinguishable I-CeuI cleavage patterns after PFGE (Fig. 6), and this pattern is very similar to bacteria in the genus *Klebsiella *[27]; no difference in cleavage pattern by SpeI, XbaI or AvrII was seen either among the seven strains (data not shown). Comparisons of 16S rRNA sequence of S1 with those of sequenced bacterial genomes in Genbank revealed close phylogenetic relatedness of S1 to *Klebsiella *strains; the 16S rRNA sequence has been deposited to Genbank with the accession number of GQ464976. Genomic analysis is under way to determine the identity and relative abundance of all members in END-49.

## Discussion

The likely health values of enterolignans and, on the other hand, difficulties in its large scale industrial production at low cost and without environmental pollution call for biotransformation technologies to convert plant lignans to them. Numerous bacterial isolates that can conduct the biotransformation have been reported [8,10,12,14-20,23]. However, most of the reported bacteria require strict anaerobic conditions to grow and metabolize plant lignans to produce enterolignans, which significantly restricts large scale production. Here in this study, we report highly efficient production of END from defatted flaxseeds through biotransformation by human intestinal bacteria without having to culture the bacteria under anaerobic conditions. The method described here has four advantages. First, instead of pure lignans (SDG, SECO, MAT, etc.), defatted flaxseed flour was used as the substrate for END production. As flaxseeds are widely available around the world and the defatted by-products of flaxseeds are usually used as animal feeds or even treated as waste, our study provides a very economic and eco-friendly method of END production using these low cost materials. Second, the high efficiency of END production by our bacterial culture system without the need of strictly anaerobic conditions makes large scale production much easier. Third, no extra carbon source would be needed in the culture, which is especially advantageous, because the most energy-efficient carbon sources, e.g., glucose, normally repress the utilization of other energy sources by microorganisms. Therefore, in the absence of common carbon sources, the biotransformation of flaxseeds into END would be remarkably enhanced. Fourth, this method is entirely harmless to the environment, as the solvents used in this procedure were only water and ethanol, both of which could be recycled.

In this study, a bacterial consortium, END-49, was obtained from human intestinal microbiota through successive subcultures. END-49 was highly efficient in converting flaxseed lignans into END, producing up to 3.9 mg g^-1^, much higher than previously reported 0.6 mg g^-1 ^(such as in [8]). END-49 consists of at least five genomically different bacterial lineages as estimated on the basis of PFGE analysis. As none of the single-colony isolated bacterial strains could produce END, we postulate that the biotransformation was conducted jointly by several different bacteria, including some or all the PFGE-resolved Group I-V strains and possibly some bacteria that escaped detection in this study. The Next-Generation sequencing technologies (e.g., 454 and SOLiD) may eventually help identify the END-producers by determining the whole genome sequences of all bacteria in the consortium and facilitate the elucidation of the pathways of END production by these bacteria.

END and ENL have two enantiomeric mirror image forms, which can be inter-converted by intestinal bacteria. In our study, END produced by "END-49" was (+)-form, consistent with the published work [18] in which SDG from flaxseed was transformed to (+)-ENL *via *(+)-SECO. Additionally, researchers have confirmed that the absolute configurations at C-2 and C-3 of END and ENL were not changed during the microbial metabolism [22]. Therefore, obviously, in our study, SDG was converted to (+)-END by human intestinal microbiota *via *(+)-SECO as a metabolic intermediate.

The method described in this study had been optimized and could be used to obtain bacterial consortia that can convert plant lignans into END or related products. Using this method, we screened fecal specimens from 28 young adults and detected END or its dehydrogenized product in all cases (data not shown), consistent with previous reports that bacteria that can convert plant lignans into END or related products are common members of the human intestinal microbiota [28,29] and they are readily obtainable for use in the bio-production of END.

## Conclusion

Biotransformation is a very economic, efficient and environmentally friendly way of mass-producing enterodiol from defatted flaxseeds.

## Methods

### Chemicals and reagents

HPLC-grade acetonitrile was purchased from Merck KGaA Co. Ltd (Darmstadt, Germany), and purified water was provided by Hangzhou Wahaha Co. Ltd (Zhejiang, China). Analytical-grade methanol, *n*-butanol, petroleum ether, ethanol, KH_2_PO_4 _and K_2_HPO_4 _were purchased from Beijing Chemical Reagents Co. Ltd (Beijing, China). Enterodiol Standard was purchased from Sigma Chemical Co. (St. Louis, MO., USA). Amberlite XAD-2 macroporous resin (20-60 mesh size, 330 m^2 ^g^-1 ^average surface area) was purchased from Supelco, Sigma-Aldrich Co. Ltd (Bellefonte, USA). Optical rotations were measured in MeOH solutions with a DIP-360 automatic polarimeter (Jasco Co., Tokyo) at 25°C, and CD spectra were determined with a JASCO J 805 spectropolarimeter (Jasco Co.).

### Plant materials

Flaxseed samples were collected from Bei-An County of Heilongjiang Province, China, and were identified as the dried seeds of *Linum usitatissimum *L. by author. Voucher specimens (sample no. 071024) were deposited was deposited in the herbarium of pharmacognosy research group, School of Pharmaceutical Sciences, Peking University Health Science Center. They were ground into powder (pass 40 mesh sieve) and then defatted by petroleum ether prior to use.

### Culture media and bacterial culture

Cooked meat medium base and Luria-Bertani (LB) nutrient agar were purchased from Beijing Land Bridge technology Co. Ltd (Beijing, China). Medium A contained tryptone 30 g, yeast extract 5 g, beef powder 5 g, glucose 3 g, NaH_2_PO_4 _5 g and amidulin 2 g, and the volume was made up to 1 liter with distilled water. Medium B was designed to lack any carbon source in the medium except defatted flaxseeds (see below), containing the following reagents (in one liter): NaCl 3 g, KH_2_PO_4 _2.6 g, K_2_HPO_4 _1.85 g, 1% (v/v) reducing solution (30 g/l L-aminothiopropionic acid and 30 g/l sodium hyposulfite, dissolved in PBS), and 1 g NH_4_Cl. Medium C was the same as medium B except the absence of any nitrogen source.

Culture was conducted as follows: 0.3 g of defatted flaxseeds was added into each of tubes containing either medium A, B or C (3 ml), which were then sealed with liquid paraffin and autoclaved at 121°C for 15 min. Into the medium, 0.3 g of fresh human feces was added and incubated at 37°C for 72 h. Supernatant of the cultures was then inspected for the appearance of END.

### Collection and processing of fecal samples

Initially, fresh fecal specimens (ca. 4.0 g each), obtained from 28 healthy young subjects (fourteen females and fourteen males, 22-33 years old), were suspended in 20 ml sterile phosphate buffer saline (PBS, 2.6 g l^-1 ^KH_2_PO_4_, 1.85 g l^-1 ^K_2_HPO_4_, PH 7.4) and 2 ml such fecal suspension was transferred to 20 ml medium, followed by incubation at 37°C for 36 h. During the fecal collection and culture preparation, no strictly anaerobic techniques or instruments were used. The fecal specimen that we used for END production was from a 33 years old female.

### High-performance liquid chromatography (HPLC)

The HPLC system consisted of Agilent 1200 series HPLC apparatus (Agilent Technologies, USA), including high-pressure binary-gradient solvent-delivery pump, DAD detector, autosampler, thermostat column compartment and chemstation (9.01 edition). Zorbax SB-C18 column (4.6 mm × 250 mm, 5 μm) was used to analyze all of the samples. Mobile phase consisted of water (A) and acetonitrile (B) in a linear gradient change from 100% A to 50% A and 50% B in 30 min. Detection wavelength was 280 nm, and the temperature of the column oven was 25°C with a flow rate of 1.0 ml min^-1^.

### Calibration of the END and SECO curves

The stock solutions of END standard (1.98 mg ml^-1^) and SECO standard (175.5 μg ml^-1^) were prepared by accurately weighing and transferring each of them into a volumetric flask (1 ml) and dissolving it in methanol. Solutions for END calibration (0.0198 ~ 1.98 mg ml^-1^) and SECO calibration (175.5 ~ 2.74 μg ml^-1^) were prepared by dilution of the stock solutions with methanol, with six dilution series being analyzed (1.98, 0.99, 0.396, 0.198, 0.099, 0.0198 mg ml^-1^) for END calibration and seven dilution series being analyzed (175.5, 87.75, 43.86, 21.94, 10.97, 5.48, 2.74 μg ml^-1^) for SECO calibration. For each calibration curve, independent dilutions were analyzed. The calibration equation of END was obtained by plotting HPLC peak areas (Y) versus the concentration of calibrators (X, mg ml^-1^), which was as follows: Y = 4433.46 X + 63.86 (R^2 ^= 0.9999), with a good linearity over the range from 0.0198 mg ml^-1 ^to 1.98 mg ml^-1^, and the calibration equation of SECO was obtained by plotting HPLC peak areas (Y) versus the concentration of calibrators (X, μg ml^-1^), which was as follows: Y = 12.59 X - 1.40 (R^2 ^= 0.9998), with a good linearity over the range from 2.74 μg ml^-1 ^to 175.5 μg ml^-1^.

### Limits of detection and quantification

Stock solutions of END and SECO standards were separately diluted to make a series of solutions with methanol and analyzed by HPLC. On the basis of signal-to-noise ratio (S/N), the limits of detection (LOD) and quantification (LOQ) of END standard were determined to be 0.699 μg ml^-1 ^(S/N = 3) and 1.398 μg ml^-1 ^(S/N = 10), respectively. The LOD and LOQ of SECO standard were determined to be 0.690 μg ml^-1 ^(S/N = 3) and 1.370 μg ml^-1 ^(S/N = 10), respectively.

### Sampling of the cultures

A volume of 200 μl of culture was sampled every 24 h and extracted with 400 μl *n*-butanol saturated with water. A portion of *n*-butanol extracts (320 μl) was transferred to a centrifuge tube and evaporated to dryness by N_2_. The residue was dissolved in 200 μl methanol and centrifuged for 3 min (12500 r min^-1^), and then 20 μl of the supernatant was filtered and analyzed by HPLC.

### Successive passages of cultures for sustained production of END

A culture was started with a fecal specimen at 37°C and sampled every 24 hours for analysis by HPLC. As END could be detected in the culture as early as within the first 24 hours at concentrations of 31.45 ± 1.51 mg l^-1 ^and the yields remained relatively stable for 6 days (starting to decline on day 9; data not shown), we used an interval of 6 days for successive passages of the culture by 1:10 dilutions in medium B without paraffin, as strict anaerobic culture conditions were not necessary (see above). A portion of the first fecal culture was stocked on day 6 from the initiation of the culture in 25% (v/v) glycerol at -80°C as "passage 1" (designated as END-1); a portion of each of all successive subcultures was stocked on the 6th day of the culture in the same way and was designated as END-2, END-3, and so on. To identify the bacteria that were involved in the biotransformation of flaxseed lignans into END, we first needed to select them out of the initial bacterial mixture in the fecal specimen. Our general strategy was to dilute the cultures in which END was produced and use the highest dilution of the bacterial culture that still produced END for successive passages in medium B, which would support only the bacteria that use defatted flaxseeds as a carbon source.

### Pulsed field gel electrophoresis (PFGE)

The endonucleases I-CeuI, AvrII, XbaI and SpeI were purchased from New England Biolabs. PFGE was performed in a CHEF - DRII system (Bio-Rad). Preparation and digestion of high molecular weight genomic DNA, digestion of DNA in agarose blocks and separation of DNA by PFGE, were as reported [30,31].

## Authors' contributions

CZW, ZRG, GRL, GXZ, JT and YNZ cultured bacteria from the human fecal samples, optimized culture conditions, and characterized and stocked the bacteria; CZW isolated the bacteria, carried out 16S rRNA sequence analysis on the bacteria and submitted the sequence to Genbank; XQM detected production of END, ENL, SECO, SDG, etc., and extracted, purified and characterized these products; MM participated in the detection of products; XQM and DHY drafted the manuscript; SQC and BSK provided equipment and reagents; DHY and SLL designed and supervised the project; SLL wrote the final manuscript. All authors read and approved the final manuscript.
